# Phenotype-Specific Response of Circulating miRNAs Provides New Biomarkers of Slow or Fast Muscle Damage

**DOI:** 10.3389/fphys.2018.00684

**Published:** 2018-06-05

**Authors:** Julien Siracusa, Nathalie Koulmann, Antoine Sourdrille, Charles Chapus, Catherine Verret, Stéphanie Bourdon, Marie-Emmanuelle Goriot, Sébastien Banzet

**Affiliations:** ^1^Unité de Physiologie de l’Exercice et des Activités en Conditions Extrêmes, Département Environnements Opérationnels, Institut de Recherche Biomédicale des Armées, Brétigny-sur-Orge, France; ^2^Ecole du Val-de-Grâce, Paris, France; ^3^Unité de Biologie Moléculaire, Département des Plateformes et Recherche Technologique, Institut de Recherche Biomédicale des Armées, Brétigny-sur-Orge, France; ^4^Bureau de Gestion de Recherche Clinique, Institut de Recherche Biomédicale des Armées, Brétigny-sur-Orge, France; ^5^Unité de Thérapie Tissulaire et Traumatologie de Guerre, Département Soutien Médico-Chirurgical des Forces, Institut de Recherche Biomédicale des Armées, Brétigny-sur-Orge, France; ^6^INSERM U 1197, Clamart, France

**Keywords:** biomarkers, circulating miRNAs, muscle damage, muscle fiber type, rat, EIMD, statins, myosin heavy chain

## Abstract

Skeletal muscle is a heterogeneous tissue composed of a continuum of contracting fibers ranging from slow-type to fast-type fibers. Muscle damage is a frequent event and a susceptibility of fast-fibers to exercise-induced damage (EIMD) or statins toxicity has been reported. Biological markers of muscle damage such as creatine kinase (CK) are not fiber-type specific and new biomarkers are needed. Some microRNAs (miRNAs) are specific to the muscle tissue, can be found in the extracellular compartment and can rise in the plasma following muscle damage. Our aim was to identify whether a set of circulating miRNAs can be used as fiber-type specific biomarkers of muscle damage in a model of traumatic (crush) injuries induced either in the slow *soleus* (SOL) or in the fast *extensor digitorum longus* (EDL) muscles of rats. A subset of miRNAs composed of miR-1-3p, -133a-3p, -133b-3p, 206-3p, -208b-3p, 378a-3p, -434-3p, and -499-5p were measured by RT-PCR in non-injured SOL or EDL muscle and in the plasma of rats 12 h after damage induced to SOL or EDL. MiR-133b-3p, -378a-3p, and -434-3p were equally expressed both in SOL and EDL muscles. MiR-1-3-p and -133a-3p levels were higher in EDL compared to SOL (1.3- and 1.1-fold, respectively). Conversely, miR-206-3p, -208b-3p, and -499-5p were mainly expressed in SOL compared to EDL (7.4-, 35.4-, and 10.7-fold, respectively). In the plasma, miR-1-3p and -133a-3p were elevated following muscle damage compared to a control group, with no difference between SOL and EDL. MiR-133b-3p and -434-3p plasma levels were significantly higher in EDL compared to SOL (1.8- and 2.4-fold, respectively), while miR-378a-3p rose only in the EDL group. MiR-206-3p levels were elevated in SOL only (fourfold compared to EDL). Our results show that plasma miR-133b-3p and -434 are fast-fiber specific biomarkers, while miR-206-3p is a robust indicator of slow-fiber damage, opening new perspectives to monitor fiber-type selective muscle damage in research and clinic.

## Introduction

Skeletal muscle damage is a frequent event that occurs in a wide range of situations such as intense/prolonged or unaccustomed exercise but also following trauma, drug use, extreme heat exposure, virus and bacterial infections and neuromuscular diseases (Khan, 2009; Taha et al., 2014). Clinical symptoms are sometimes difficult to assess and diagnosis is performed by monitoring biological markers such as serum creatine kinase (CK) or myoglobin (Sorichter et al., 1999; Ohlendieck, 2013). Although they are widely used both in research and clinic, these biomarkers suffer significant limitations such as high interindividual variability and low specificity (Sorichter et al., 1999; Brancaccio et al., 2007).

MicroRNAs (miRNAs) are small non-coding RNAs involved in the post-transcriptional regulation of gene expression (Chang and Mendell, 2007). MiRNAs can be secreted by - or can leak out of injured cells and can be measured in various biofluids, including blood (Turchinovich et al., 2012). Some miRNAs are ubiquitously expressed across tissues or cell types, while others are highly tissue-specific (Landgraf et al., 2007; Lee et al., 2008). MyomiRs (namely miR-1, -133a, -133b, -206, -208a, -208b, -486, -499) are a class of muscle and/or cardiac specific miRNAs. They are involved in numerous cellular processes such as myoblast proliferation and differentiation, muscle regeneration as well as in phenotype specification (for review, see Diniz and Wang, 2016). High levels of plasma/serum myomiRs are found in Duchenne (DMD) or Becker (BMD) muscular dystrophy patients as well as in animal models (Cacchiarelli et al., 2011; Vignier et al., 2013; Jeanson-Leh et al., 2014), and are therefore considered as new biomarkers of muscle dystrophies. In healthy organisms, our group demonstrated that exercise-induced muscle damage (EIMD) in human, as well as toxic muscle injury in rats, induced an early elevation of a subset of muscle specific (miR-1, -133a, 133b, -206, -208b, -499) and non-muscle specific miRNAs (miR-378a, -434) (Banzet et al., 2013; Siracusa et al., 2016). These results were associated with a decrease in muscle maximal voluntary contraction level, a strong indicator of muscle damage in humans (Paulsen et al., 2012), or with direct histological evidences of muscle necrosis in rats, demonstrating they are relevant biomarkers of acute muscle damage (Banzet et al., 2013; Siracusa et al., 2016).

Muscle fibers are classified according to their contractile phenotype, with slow-type (I) and fast-type (II) fibers (Schiaffino and Reggiani, 2011). The latter are further classified in IIa, IIx, and IIb in rodents; in human only IIa and IIx fibers are found. A vulnerability of fast fibers has been reported in various situations such as physical eccentric exercise (Lieber et al., 1988; Macpherson et al., 1996; Shepstone et al., 2005). A series of experiments suggest that type II fibers are more susceptible to damage due to higher strains related to a shorter length compared to type I fibers (for review, see Lieber and Friden, 1999). However, contradictory results are reported in other studies where type-I fibers seem to be preferentially damaged in eccentric exercise (Armstrong et al., 1983; Carmona et al., 2015). In medical practice, statins are widely used as cholesterol-lowering drugs. This class of molecule is known to have muscle side effects ranging from mild myalgia to life threatening rhabdomyolysis (Taha et al., 2014). These alterations appear to be more pronounced in type II fibers that in type I, possibly due to a lower mitochondrial content in fast fibers, leading to an energetic metabolism imbalance (Westwood et al., 2005; Simsek Ozek et al., 2014). Though, the ability to discriminate between damage to slow and fast fiber phenotype could be of great interest in both research and clinic. However, classical biomarkers (myoglobin and CK) are not fiber-type specific (Sorichter et al., 1999).

Interestingly, several studies have reported differential expression of muscle-specific miRNAs based on the skeletal muscle phenotype (McCarthy and Esser, 2007; van Rooij et al., 2009; Boettger et al., 2014). This was confirmed at the single myofiber level, but had only limited impact on their blood profile in a rat model of massive muscle damage (Siracusa et al., 2016). Whether these miRNAs could be useful phenotype-specific biomarkers of muscle damage in a milder and more physiological model of muscle injury remains to be determined. To address this question, we analyzed the plasma levels of these damage-responsive miRNAs after moderate traumatic injuries of the slow *soleus* or the fast EDL muscles of rats.

## Materials and Methods

### Animals

Young adult male and female (2 months) Wistar (RjHan:WI) rats were purchased from Janvier Labs (Le-Genest-Saint-Isle, France). They were housed at 22 ± 1°C, on a 12 h: 12 h light/dark cycle and provided with food and water *ad libitum*. All experiments received prior approval from the Animal Ethics Committee for Animal Research of the Service de Santé des Armées (C2EA-SSA).

### Experimental Design

Thirty-two rats were randomly distributed in four experimental groups and either received traumatic muscle injury of the slow *soleus* muscle (SOL, *n* = 8), of the fast *Extensor digitorum longus* muscle (EDL, *n* = 8), a sham surgery (SHAM, *n* = 8) or constituted a control group (CTRL, *n* = 8).

### Traumatic Muscle Injury

Rats were anesthetized with an intraperitoneal (i.p.) injection of a mixture of 60 mg kg^-1^ of ketamine (Laboratoire Renaudin, Itxassou, France) and 0.5 mg kg^-1^ of medetomidine (Elanco, Greenfield, IN, United States). Either *soleus* or EDL muscle of the left hindlimb was surgically exposed and injured by crushing with a forceps. After an incision of the skin and fasciae, the forceps was inserted along the *soleus* or EDL muscle covering the entire length of the muscles. Injury was done by a single pressure of the forceps maintained for 10 s. Then, fasciae and skin were sutured and rats received a subcutaneous injection of 0.05 mg kg^-1^ of buprenorphine (Axience, Pantin, France) to prevent post-operative pain. The same surgical gesture was applied to SHAM rats, with no muscle damage.

### Tissue Processing

All rats were anesthetized 12 h post-intervention with an i.p. injection of 150 mg kg^-1^ of pentobarbital sodium (Céva Santé Animale, Libourne, France). Then, rats were euthanatized with an exsanguination performed by a puncture of blood in the abdominal aorta artery. Whole blood was drawn into a K_2_EDTA tube (BD Vacutainer, Becton, Dickinson and Company, Plymouth, United Kingdom) centrifuged (2000 *g*, 10 min, 4°C) and plasma was transferred to a new vial and centrifuged again. *Soleus* and EDL muscles were collected, weighted and immediately frozen in liquid nitrogen and stored at -80°C.

### miRNA Extraction

Plasma miRNA was extracted as previously described in Siracusa et al. (2016). Total RNA was isolated from 100 μL of plasma with a mirVana Paris Kit (Ambion, Austin, TX, United States) according to the manufacturer’s protocole, followed by an additional precipitation step. Column elution was performed with 180 μL of hot (90°C) sterile water and 18 μL sodium acetate 3 mol L^-1^ (Sigma-Aldrich, St. Louis, MO, United States), 396 μL of ethanol (100%), and 1 μL of GlycoBlue (Ambion) were added. Total RNAs were precipitated for 20 min at -20°C and centrifuged (12,000 *g*, 15 min, 4°C). The pellet was washed with 70% ethanol, dried using a centrifugal vacuum concentrator (2 min, 45°C) and resuspended in 12 μL of sterile water.

Muscle RNA was extracted using a miRNeasy Mini Kit (Qiagen, Courtaboeuf, France) from 10 to 15 mg of muscle homogenized in 700 μL of QIAzol Lysis reagent (Qiagen) according to the manufacturer’s protocol.

### cDNA Synthesis

cDNA synthesis from plasma and muscle RNA was performed with the Universal cDNA Synthesis Kit (Exiqon, Vedbæk, Denmark). Plasma RNA was reverse transcribed from 5 μL of total RNA diluted 1:6 in a 10 μL reaction volume and muscle RNA was reverse transcribed from 5 μL of diluted RNA (5 to 10 ng μL^-1^) according to the manufacturer’s protocol.

### Real-Time Quantitative qPCR

Real-time quantitative qPCR (RT-qPCR) assays were performed on a LightCycler 480 instrument (Roche Applied Science, Manheim, Germany) with 4 μL of cDNA (diluted 1:80 in sterile water) and 6 μL of ExiLENT SYBR Green Master Mix (Exiqon) in a 10 μL reaction volume. First a quality control qPCR was performed for all samples using *Rattus norvergicus* (rno)-miR-103a-3p (Exiqon), with a quantification cycle cutoff of ≤32 (see Supplementary Figure 1). Then we measured miRNAs of interest: rno-miR-1-3p, -133a-3p, -133b-3p, -206-3p, mmu-208b-3p, rno-miR-499-5p, -378a-3p, -434-3p (Exiqon). The number of reference miRNA and their stability were determined with geNorm version 3.4 (Vandesompele et al., 2002). Final quantification was the geometrical mean of the quantification performed with each reference miRNA. A list of target and reference miRNAs, their raw quantification cycle ranges and qPCR primer information has been added for plasma and muscles in Supplementary Tables 1, 2.

Red blood cells influence on circulating miRNAs has been described and hemolysis can result in an alteration of plasmatic miRNA profiles (Pritchard et al., 2012; Kirschner et al., 2013). The influence of this parameter on the set of damage-responsive miRNAs has been assessed in our previous work where hemolysis did not resulted in alteration of their levels (Siracusa et al., 2016).

### MYH Isoform Analysis

MYH isoform analysis was performed on 10–15 mg of *soleus* or EDL muscles collected on non-injured rats. Myosin was extracted in seven volumes of buffer solution (0.3 M NaCl, 0.1 M NaH_2_PO_4_, 0.05 M Na_2_HPO_4_, 0.01 M Na_4_P_2_O_7_, 1 mM MgCl_2_-6H_2_O, 10 mM EDTA and 1.4 mM 2β-mercaptoethanol, pH = 6.5). Electrophoresis was performed using a Mini Protean II system (BioRad, Marnes-la-Coquette, France) with 8 and 4% acrylamide-bis (50:1) separating and stacking gels, respectively, containing 0.4% sodium dodecyl sulfate (SDS). Myofibril samples were denatured with the SDS incubation medium and according to the method of Laemmli. Gels were run at a constant voltage (72 V) for 31 h and then silver stained. The MYH protein isoform bands were scanned and quantified using a densitometer (ChemiDoc XRS System, Bio-Rad) (Agbulut et al., 1996).

### Muscle Histology

*Soleus* and EDL cryosections (8 μm) were performed with a cryostat (Leica Biosystems, Wetzlar, Germany) and were stained with Hematoxylin phloxine (HPS) saffron with a Varistain automaton (Thermo Scientific, Waltham, MA, United States). Images were acquired with a Leica DMI6000 B microscope (Leica Microsystems, Wetzlar, Germany). Muscle cartographies were performed with the MetaMorph software (Molecular Devices, Sunnyvale, CA, United States).

### miRNA Diagnostic Performance

The ability of circulating miRNAs to discriminate between damage originating from a slow *soleus* (SOL) or a fast *extensor digitorum longus* (EDL) muscle has been evaluated using receivers operating characteristic (ROC) curve analysis and the resulting area under the curve (AUC) using R version 3.4.3 (R Core Team, 2017). The missing data have been imputed using nearest neighbor averaging (package *impute*). A logistic regression analysis has been used to establish a miRNA combination in order to evaluate the probability for a sample to belong to SOL instead of EDL. The diagnostic accuracy of the combination was evaluated with a ROC curve and AUC.

### Statistical Analysis

Results are represented as box-and-whiskers plots. Graphics were generated using Prism software version 6.01 (GraphPad, San Diego, CA, United States). Statistical tests were performed with Statistica software version 10 (Statsoft, Maisons-Alfort, France). Plasma profiles were analyzed with a one-way ANOVA analysis and *post hoc* comparisons were performed with a Tukey HSD test. Muscle miRNAs levels were compared using a *t*-test. Results with a *P*-value ≤ 0.05 were considered significant.

## Results

### Soleus and EDL Muscle Weight

Healthy *soleus* and EDL muscles weight were similar (0.17 ± 0.03 vs. 0.19 ± 0.03 g, respectively).

### Myosin Heavy Chain Isoform Distribution

**FIGURE 1:**
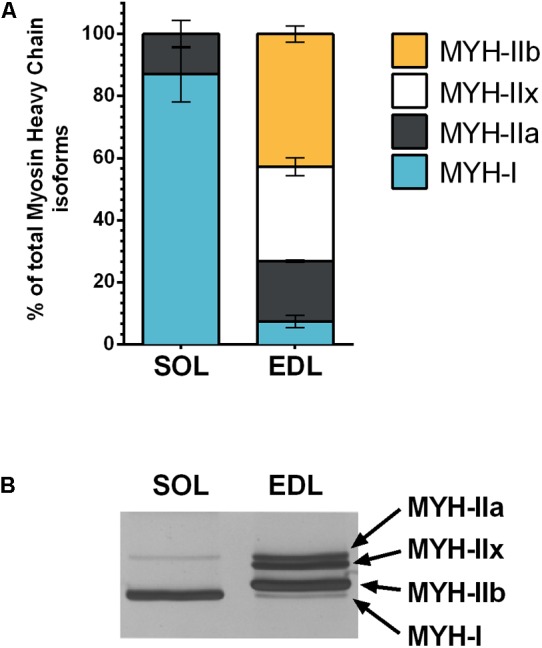
Myosin heavy chains (MYH) isoform expression in muscle tissue. Total MYH was extracted from slow-type *soleus* (SOL) or fast-type *extensor digitorum longus* (EDL) muscles, MYH isoforms were separated by SDS-PAGE and silver stained. The MYH isoforms distribution was determined; data are expressed as a percentage of total MYH **(A)**. A representative gel displaying type I, IIa, IIx, and IIb isoforms distribution is shown **(B)**. A full scan of the entire gel is given in Supplementary Figure 2.

As expected, MYH isoform analysis revealed that *soleus* muscles mainly comprised slow type MYH-I isoform (87% of total MYH) associated with a small fraction of MYH-IIa isoform (13%; **Figures 1A,B**). Conversely, EDL muscles comprised a mix of fast MYH-IIb (42.8%), MYH-IIx (30.3%), MYH-IIa (19.5%) isoforms and a small fraction MYH-I (7.4%) isoform (**Figures 1A,B**).

### Expression of Skeletal Muscle miRNA in Soleus and EDL Tissue

**FIGURE 2:**
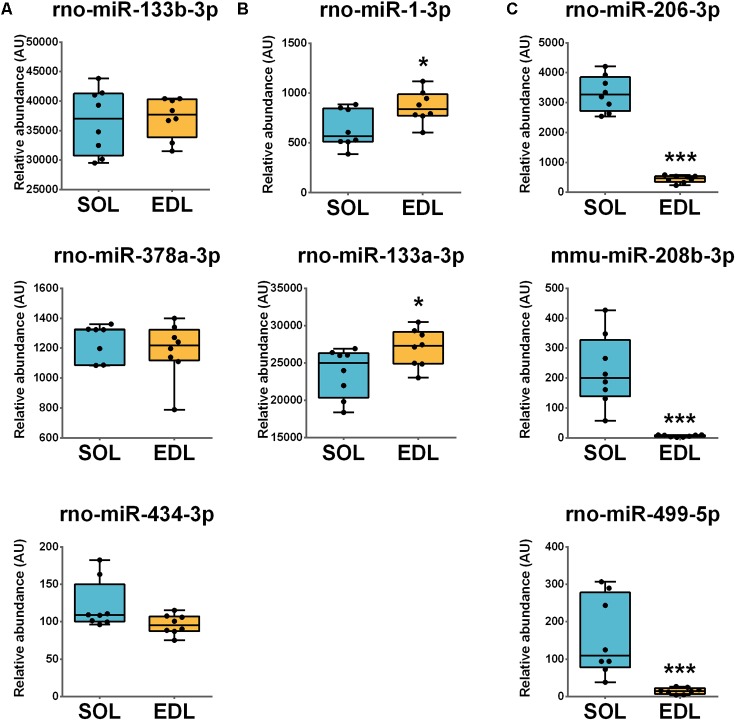
Muscle miRNAs levels are influenced by muscle phenotype. Damage-responsive miRNAs levels were measured in non-injured slow-type *soleus* (SOL) and fast-type *extensor digitorum longus* (EDL) muscles by real-time quantitative RT-PCR. Three miRNAs profiles were found with **(A)** rno-miR-133b-3p, rno-miR-378a-3p, and rno-miR-434-3p equally expressed in both muscle types, **(B)** rno-miR-1-3p and rno-miR-133a-3p with higher expression in EDL and **(C)** rno-miR-206-3p, mmu-miR-208b-3p, and rno-miR-499-5p with higher expression in SOL. Results were normalized to three reference miRNAs. Results are expressed as interquartile ranges (boxes), median (horizontal bars inside boxes), 5^th^ and 95^th^ percentiles, whiskers and individual values (dots). *n* = 8 per groups. ^∗^*P* ≤ 0.05, ^∗∗^*P* ≤ 0.01, and ^∗∗∗^*P* ≤ 0.001 versus SOL, as determined by a *t*-test. AU, relative abundance arbitrary units; *mmu, Mus musculus; rno, Rattus norvegicus*.

The muscle expression of miRNA of interest in non-injured slow *soleus* and fast EDL muscles resulted in the identification of three distinct profiles. A subset of miRNAs, miR-133b-3p, -378a-3p, and -434-3p, was equally expressed both in SOL and EDL muscles (**Figure 2A**). MiR-1-3-p and -133a-3p levels were significantly higher in the EDL compared to the *soleus* (1.3- and 1.1-fold, respectively; **Figure 2B**). Conversely, miR-206-3p, miR-208-3p, and -499-5p expression was significantly higher in the *soleus* muscle compared to the EDL (7.4-, 10.7-, and 35.4-fold respectively; **Figure 2C**).

### Muscle Histological Examination

**FIGURE 3:**
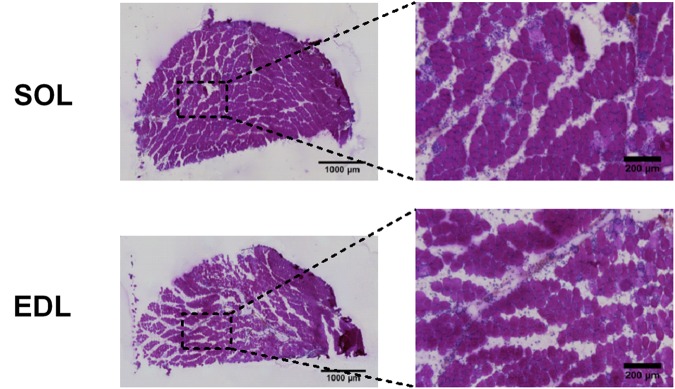
Representative muscle sections following traumatic damage of a slow or a fast-type muscle. Muscle cryosections were stained with hematoxylin phloxine saffron of the slow *soleus* (SOL) and fast *extensor digitorum longus* (EDL) muscles 12 h after crushing. Muscle fascicules are separated by an important edema. Necrotic (pale) fibers and a mononuclear cell infiltrate are visible.

Traumatic muscle damage examined by HPS-staining 12 h following crushing were substantial both in *soleus* and EDL muscles. Damaged muscles were mainly characterized by the separation of the fibers fascicles and presence of an edema with moderate fiber necrosis (**Figure 3**). Infiltration of mononuclear cells was also observed around and inside necrotic fibers both in *soleus* and EDL.

### Circulating miRNAs Profiles Following Traumatic Damage of *Soleus* and EDL Muscles

**FIGURE 4:**
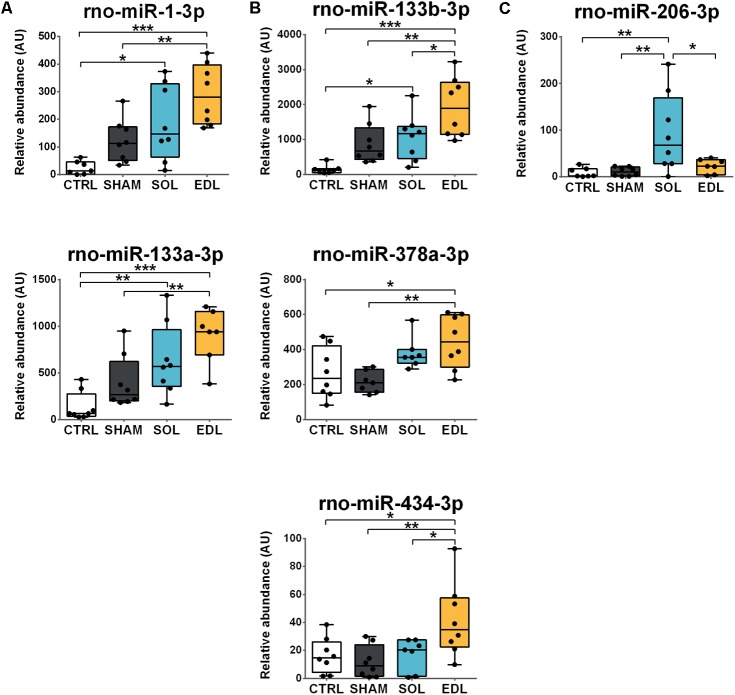
Circulating miRNAs levels following traumatic damage of a slow or a fast-type muscle. Damage-responsive miRNAs levels were measured in the plasma of control rats (CTRL), 12 h following sham-operation (SHAM) or traumatic muscle damage of slow-type *soleus* (SOL) and fast-type *extensor digitorum longus* (EDL) muscles. Three miRNAs profiles were found with **(A)** rno-miR-1-3p and rno-miR-133a-3p rising in response to both SOL and EDL muscle damage, **(B)** rno-miR-133b-3p, rno-miR-378a-3p, and rno-miR-434-3p with higher levels following EDL muscle damage and **(C)** rno-miR-206-3p with higher levels following SOL muscle damage. MiRNAs plasma levels were measured by real-time quantitative RT-PCR and results were normalized to six reference miRNAs. Results are expressed as interquartile ranges (boxes), median (horizontal bars inside boxes), 5^th^ and 95^th^ percentiles, whiskers, and individual values (dots). *n* = 8 per groups. Statistical differences between groups are represented by horizontal bars above boxes and are ^∗^*P* ≤ 0.05, ^∗∗^*P* ≤ 0.01, and ^∗∗∗^*P* ≤ 0.001, as determined with a one-way analysis of variance test and a subsequent Tukey HSD *post hoc* test. AU, relative abundance arbitrary units; *mmu, Mus musculus; rno, Rattus norvegicus*.

Three different circulating miRNAs profiles were observed 12 h following muscle damage of slow or fast muscles. MiR-1-3p and -133a-3p plasma levels were elevated following muscle damage compared to CTRL, with no difference between SOL and EDL (**Figure 4A**). MiR-133b-3p and -434-3p plasma levels were significantly higher in EDL compared to SHAM, CTRL as well to SOL (1.8- and 2.4-fold, respectively), while miR-378a-3p rose only in the EDL group compared to both SHAM and CTRL (**Figure 4B**). MiR-206-3p levels were significantly elevated in SOL compared to the other groups (fourfold compared to EDL; **Figure 4C**). MiR-208b-3p and -499-5p remained undetectable in most of our samples.

**FIGURE 5:**
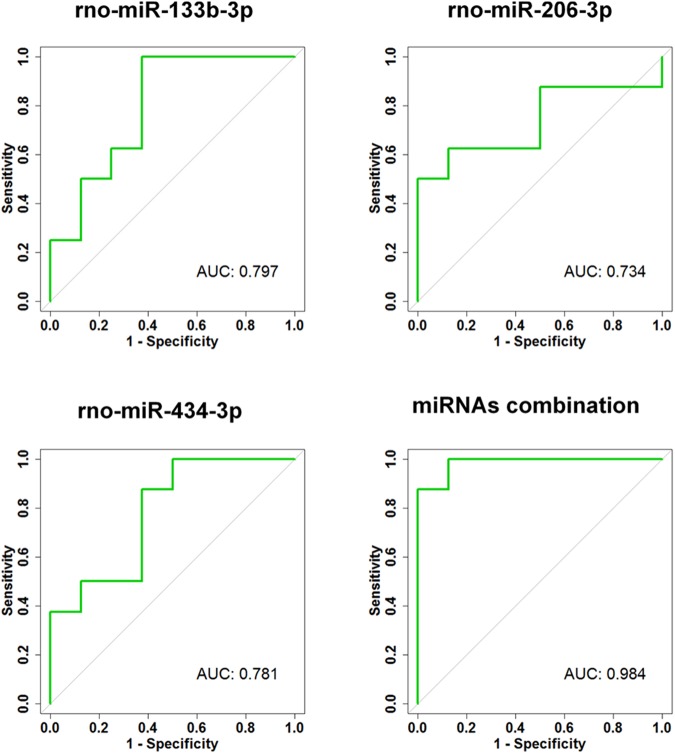
Individual and combined diagnostic accuracy of circulating miRNAs. The ability of circulating rno-miR-133b-3p, rno-miR-206-3p, and rno-miR-434-3p to discriminate between damage originating from a slow-type *soleus* (SOL) or a fast-type *extensor digitorum longus* (EDL) muscle has been evaluated using receiver operating characteristic curve and the corresponding area under the curve (AUC). A logistic regression analysis has been used in order to evaluate whether a combination of these three circulating miRNAs could increase the ability to discriminate between SOL and EDL; *rno, Rattus norvegicus*.

The ability of a miRNA to discriminate between a damage originating from a slow (SOL) or a fast (EDL) muscle has been evaluated using a ROC curve analysis. MiR-133b-3p, -434-3p and -206-3p, that were significantly increased in either SOL or EDL compared to the other injured group were used for this analysis. As revealed by their AUC, miR-133b-3p (AUC = 0.781), -206-3p (AUC = 0.734), and -434-3p (AUC = 0.797) individual diagnostic accuracy were moderate (**Figure 5**). Therefore, we used a logistic regression analysis in order to evaluate whether a combination of these three circulating miRNAs could increase the ability to discriminate between SOL and EDL. The miRNAs combination substantially increased the ability to discriminate between SOL and EDL as illustrated by the AUC = 0.984 (**Figure 5**).

## Discussion

In the present work, we aimed at determining whether a subset of damage responsive miRNAs could be useful phenotype-specific biomarkers of muscle fiber damage in a physiological model of muscle injury in rats. We found that circulating muscle-specific miRNA profiles are different in response to slow or fast muscle damage. Despite similar muscle weight and damage, plasma miR-206-3p was higher in response to *soleus* damage whereas miR-133b-3p and miR-434-3p were higher after EDL damage.

As previously reported, myomiRs expression differs between slow and fast-type muscles (McCarthy and Esser, 2007; van Rooij et al., 2009; Endo et al., 2013; Muroya et al., 2013; Boettger et al., 2014; Zhang et al., 2014). First, miRNAs such as miR-1-3p and -133a-3p expression was higher in the fast EDL than slow *soleus*. These profiles are consistent with previous results obtained in a variety of muscles and species (Endo et al., 2013; Muroya et al., 2013; Boettger et al., 2014; Zhang et al., 2014). For instance, Muroya et al. (2013), described higher levels of miR-1 and -133a in the *semitendinous* (fast) compared to the *masseter* (slow) muscle of Japanese Black cattle (Muroya et al., 2013). Second, miR-206-3p, -208b-3p, and -499-5p were mainly or exclusively expressed in the slow *soleus* muscle. Higher expressions of these miRNAs in slow muscles are reported (McCarthy and Esser, 2007; van Rooij et al., 2009; Boettger et al., 2014). Furthermore, their distribution was consistent with our previous results in rat single fibers, suggesting they are mainly produced in myofibers and that the other cell types do not significantly contribute to expression in muscle tissue (Siracusa et al., 2016). Due to the fact that *soleus* and EDL have similar weights, that the surgical approach is the same and the crush applied the same way, it is likely that the differences in circulating miRNA response are due to this phenotype-related pattern of expression within muscles.

Both miR-208b-3p and -499-5p are expressed almost exclusively in *soleus*, consistent with the location of their coding DNA within Myh7 (coding for MYH-1) and Myh7b (coding slow-tonic MYH transcribed in slow fibers) (van Rooij et al., 2009). They were not detectable in the plasma of control rats as reported in most species studied, including human (Aoi et al., 2013; Banzet et al., 2013; Muroya et al., 2013; Siracusa et al., 2016). We did not observe any increase of the circulating levels of these myomiRs following traumatic muscle damage. Although no reliable comparison of miRNA abundance can be made with RT-PCR, it should be noted that the mean quantification cycles for miR-208b-3p and -499-5p (24.35 and 25 respectively) measured in the *soleus* muscle are high compared to the other miRNAs (from 16.2 for miR-133b-3p to 22.54 for miR-1-3p), suggesting they are less abundant. This is further supported by the study of Muroya et al. (2013), where the sequencing read count of bta-miR-1, -133a and -206 are among the most frequently detected miRNAs in bovine skeletal muscles (mean read count of both *semitendinous* and *masseter* muscles ranging from ∼1260528 for bta-miR-1 to ∼80481 for bta-miR-206), while bta-miR-208b was detectable but with lower read count (∼1687) (Muroya et al., 2013). Surprisingly, in our previous study we observed that miR-208b-3p and -499-5p were significantly elevated following massive muscle damage induced by a myotoxic injection. Thus, it is possible that the milder damage induced by crush may not be sufficient to elicit a significant increase of their plasmatic levels (Siracusa et al., 2016).

The other damage-responsive myomiRs, miR-1-3p, -133a-3p and -206-3p, were robustly detectable in the plasma following traumatic muscle damage. We observed a significant increase in the circulating level of miR-206-3p in response to *soleus* damage whereas miR-133b-3p was higher after EDL damage. This may be surprising because these myomiRs are reported to be coded in a cluster and transcribed in a bicistronic transcript (van Rooij et al., 2008). Yet, our results in both muscle tissue and plasma suggest that they are expressed separately. Accordingly, Cesana et al. (2011) suggested that miR-133b and -206 are coded in an intron and an exon of linc-MD1, a highly conserved muscle-specific long non-coding RNA (Cesana et al., 2011). MiR-206 appears to be autonomously transcribed with its own promoter while miR-133b is co-transcribed with the linc-MD1 RNA.

Elevated circulating myomiRs levels have been described in patients with Duchenne muscular dystrophy (DMD), a pathology characterized by the loss of dystrophin protein leading to cycles of degeneration and regeneration of the muscle tissue (Cacchiarelli et al., 2011; Zaharieva et al., 2013; Jeanson-Leh et al., 2014). As for EIMD and statins treatment, it is reported that fast fibers are preferentially affected (Webster et al., 1988; Li et al., 2014), leading to a progressive decrease of fast fibers in the muscle of 2 to 6 years old patients (Li et al., 2014). Interestingly, the slow-fiber enriched myomiRs miR-206, -208b, and -499 were found to be positively correlated with the percentage of type I fiber in DMD patients older than 6 years (Li et al., 2014). Our results are in general agreement with these observations and support the fact that some circulating miRNAs can be used to monitor muscle damage originating from slow or a fast myofibers. The classical biomarkers such as CK or myoglobin are not able to inform on the phenotype of injured fibers (Chapman et al., 2013), and the identification of fiber-type specific biomarkers of muscle damage can be useful in research and clinic. Several studies have been conducted to identify such specific biomarkers. The group of Nosaka studied the serum slow (ssTnI) and fast skeletal troponin I (fsTnI), exclusively expressed in the slow and fast muscles, respectively (Chapman et al., 2013). Young men performed numerous eccentric contractions of the elbow flexors in order to induce EIMD. The exercise was associated with a decrease in force production and an increase in serum CK activity peaking 4 days following muscle damage. The fsTnI level increased with the same time-course, while ssTnI did not change, suggesting fsTnI may reflect muscle damage to type-II fibers (Chapman et al., 2013). In a similar experiment, Carmona et al. (2015), measured fast (FM) and slow myosin (SM) isoforms in the serum after EIMD was provoked by a repetition of half-squat in young men. In agreement with the study of Chapman et al. (2013), fast-fiber specific biomarker FM was elevated 48 h later, while slow-fiber SM remained unchanged. In the present study, we identify microRNAs specifically elevated following damage to a slow or a fast muscle. These miRNAs were measured 12 h following the crush injury because our previous work identified this time point as the peak of detection of circulating miRNAs following muscle damage (Siracusa et al., 2016). Compared to the results obtained with muscle protein assessment, miRNAs allow an early diagnosis, in less than a day (Chapman et al., 2013; Carmona et al., 2015; Siracusa et al., 2016). Furthermore, while we showed that the ability of these circulating miRNAs to discriminate between damage originating from a slow or a fast muscle was moderate, the combination of three miRNAs was able to discriminate SOL and EDL almost perfectly. We therefore think that this early response added to high diagnostic accuracy is very complementary to other fiber-type specific biomarkers since it could help detect muscle damage earlier.

## Conclusion

Taken together, our results demonstrate that some circulating miRNAs can be useful to identify muscle damage originating from fast or slow muscle fibers in rats. These new biomarkers open new perspectives, because they are complementary to protein fiber-type specific biomarkers and rise early in the plasma, allowing for a rapid diagnosis. The medical field may benefit of these biomarkers, as they might provide information on EIMD or statins side effect but it remains to be determined in future studies in human.

## Author Contributions

JS, NK, and SéB designed the study. JS, NK, AS, StB, M-EG, and SéB participated in the data collection. JS, NK, AS, CC, CV, and SéB analyzed and interpreted these data. JS and SéB drafted the article. NK and SéB critically revised the article. All authors gave their final approval for this version of the article for publication.

## Conflict of Interest Statement

The authors declare that the research was conducted in the absence of any commercial or financial relationships that could be construed as a potential conflict of interest.
