# Supplementary material for: Phenotype-Specific Response of Circulating miRNAs Provides New Biomarkers of Slow or Fast Muscle Damage
Source: Front Physiol. 2018 Jun 5;9:684. doi: 10.3389/fphys.2018.00684 (PMC5996145; doi:10.3389/fphys.2018.00684)
Supplement: Supplementary file 1 [file Table_1.DOCX]

Supplementary Material

Phenotype-specific response of circulating miRNAs provides new biomarkers of slow or fast muscle damage

**Julien SIRACUSA^*^, Nathalie KOULMANN, Antoine SOURDILLE, Charles CHAPUS, Catherine VERRET, Stéphanie BOURDON, Marie-Emmanuelle GORIOT, and Sébastien BANZET**

*** Correspondence:** Dr. Julien SIRACUSA: siracusa.julien@gmail.com

# Supplementary Table 1

Supplementary Table 1: List of target and reference microRNAs measured in plasma

| microRNA | Category | Raw Cq  (mean ± SD) | Range Cq  (min - max) | Exiqon PCR primer, Product Number | miRBase accession number |
| --- | --- | --- | --- | --- | --- |
| rno-miR-1-3p | target | 33.19 ± 2.19 | 30.38 - 40 | 205104 | MIMAT0003125 |
| rno-miR-133a-3p | target | 31 ± 1.95 | 27.95 - 35.53 | 204788 | MIMAT0000839 |
| rno-miR-133b-3p | target | 30.07 ± 1.8 | 27.46 - 34.87 | 204162 | MIMAT0003126 |
| rno-miR-206-3p | target | 36.06 ± 3.15 | 29.73 - 40 | 205642 | MIMAT0000879 |
| mmu-miR-208b-3p | target | ND | ND | 204636 | MI0005552 |
| rno-miR-499-5p | target | ND | ND | 205935 | MIMAT0003381 |
| rno-miR-378a-3p | target | 31.18 ± 1.54 | 27.98 - 33.93 | 204179 | MIMAT0003379 |
| rno-miR-434-3p | target | 36.11 ± 2.34 | 32.54 - 40 | 205190 | MIMAT0005315 |
| rno-miR-20a-5p | reference | 28.27 ± 1.32 | 25.14 - 30.33 | 204292 | MIMAT0000602 |
| rno-miR-103-3p | reference | 30.12 ± 1.45 | 27.19 - 32.75 | 204063 | MIMAT0000824 |
| rno-miR-185-5p | reference | 31.49 ± 1.71 | 27.34 - 34.21 | 204475 | MIMAT0000862 |
| rno-miR-21-5p | reference | 27.41 ± 1.47 | 25.01 - 31.18 | 204230 | MIMAT0000790 |
| rno-miR-126a-3p | reference | 29.08 ± 1.31 | 25.48 - 31.11 | 204227 | MIMAT0000832 |
| rno-miR-192-5p | reference | 29.77 ± 1.14 | 27.77 - 32.58 | 204099 | MIMAT0000867 |

Cq: qPCR quantification cycle; ND: non detectable.
