# Supplementary material for: Phenotype-Specific Response of Circulating miRNAs Provides New Biomarkers of Slow or Fast Muscle Damage
Source: Front Physiol. 2018 Jun 5;9:684. doi: 10.3389/fphys.2018.00684 (PMC5996145; doi:10.3389/fphys.2018.00684)
Supplement: Supplementary file 2 [file Table_2.DOCX]

Supplementary Material

Phenotype-specific response of circulating miRNAs provides new biomarkers of slow or fast muscle damage

**Julien SIRACUSA^*^, Nathalie KOULMANN, Antoine SOURDILLE, Charles CHAPUS, Catherine VERRET, Stéphanie BOURDON, Marie-Emmanuelle GORIOT, and Sébastien BANZET**

*** Correspondence:** Dr. Julien SIRACUSA: siracusa.julien@gmail.com

# Supplementary Table 2

Supplementary Table 2: List of target and reference microRNAs measured in *soleus* and *extensor digitorum longus* muscles

| microRNA | Category | Raw Cq  (mean ± SD) | Range Cq  (min - max) | Exiqon PCR primer, Product Number | miRBase accession number |
| --- | --- | --- | --- | --- | --- |
| rno-miR-1-3p | target | 22.74 ± 0.75 | 21.05 - 23.82 | 205104 | MIMAT0003125 |
| rno-miR-133a-3p | target | 17.22 ± 0.63 | 15.76 - 17-91 | 204788 | MIMAT0000839 |
| rno-miR-133b-3p | target / reference for rno-miR-378a-3p quantification | 16.63 ± 0.69 | 14.97 - 17.53 | 204162 | MIMAT0003126 |
| rno-miR-206-3p | target | 21.97 ± 2.2 | 18.73 - 25.4 | 205642 | MIMAT0000879 |
| mmu-miR-208b-3p | target | 27.84 ± 3.87 | 22.51 - 34.27 | 204636 | MI0005552 |
| rno-miR-499-5p | target | 27.28 ± 2.71 | 22.79 - 31.62 | 205935 | MIMAT0003381 |
| rno-miR-378a-3p | target / reference for rno-miR-133b-3p quantification | 20.6 ± 0.69 | 19.29 - 21.78 | 204179 | MIMAT0003379 |
| rno-miR-434-3p | target | 25.73 ± 0.79 | 24.31 - 26-97 | 205190 | MIMAT0005315 |
| rno-miR-103-3p | reference | 23.98 ± 0.65 | 23.05 - 25.45 | 204063 | MIMAT0000824 |
| rno-miR-26a-5p | reference | 21.27 ± 0.74 | 20.24 - 22.67 | 205905 | MIMAT0000796 |

Cq: qPCR quantification cycle
