# Supplementary figures and images for: Phenotype-Specific Response of Circulating miRNAs Provides New Biomarkers of Slow or Fast Muscle Damage
Source: Front Physiol. 2018 Jun 5;9:684. doi: 10.3389/fphys.2018.00684 (PMC5996145; doi:10.3389/fphys.2018.00684)

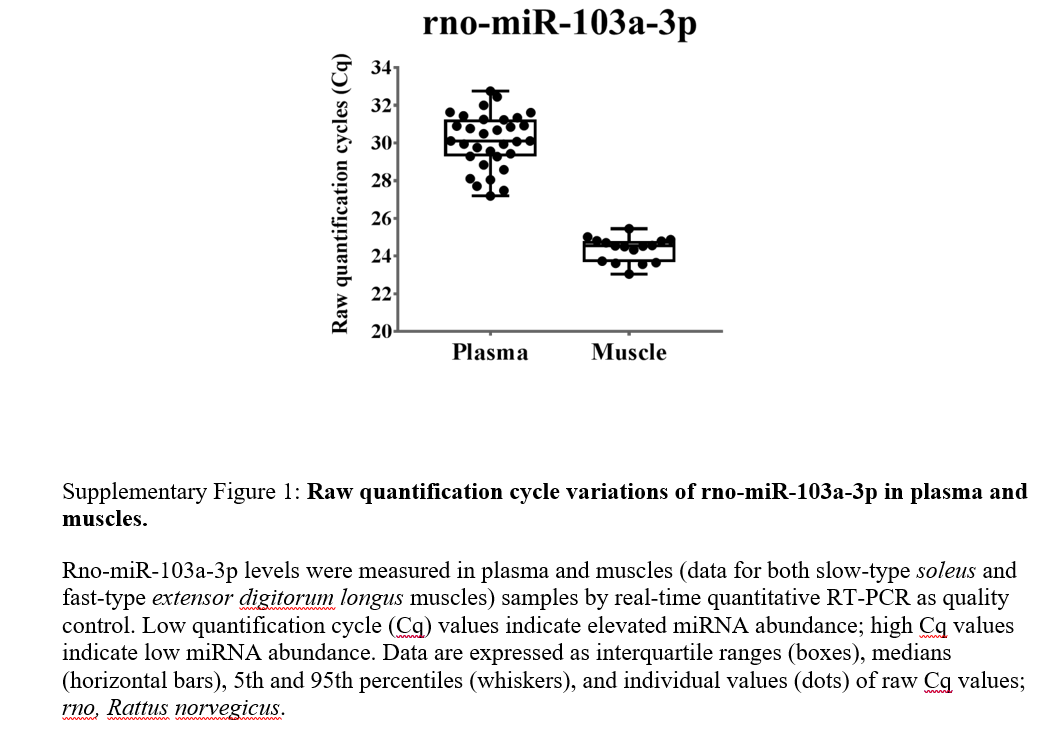

Supplement: Supplementary file 3 [file Image_1.PNG]

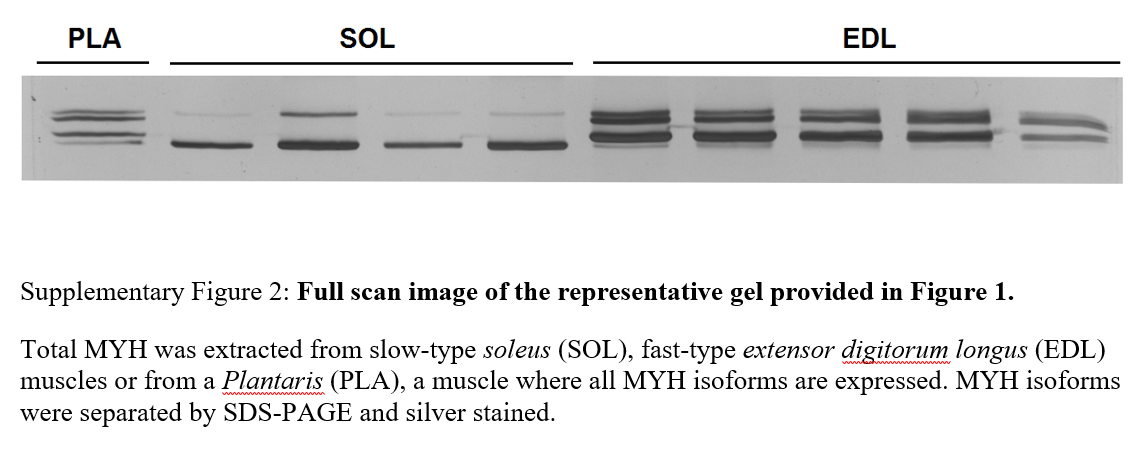

Supplement: Supplementary file 4 [file Image_2.PNG]
